# Targeting human apurinic/apyrimidinic endonuclease 1 (APE1) in phosphatase and tensin homolog (PTEN) deficient melanoma cells for personalized therapy

**DOI:** 10.18632/oncotarget.1926

**Published:** 2014-04-27

**Authors:** Rachel Abbotts, Rosalyn Jewell, Jérémie Nsengimana, David J Maloney, Anton Simeonov, Claire Seedhouse, Faye Elliott, Jon Laye, Christy Walker, Ajit Jadhav, Anna Grabowska, Graham Ball, Poulam M Patel, Julia Newton-Bishop, David M Wilson, Srinivasan Madhusudan

**Affiliations:** ^1^ Academic Unit of Oncology, Division of Cancer and Stem Cells, School of Medicine, University of Nottingham, Nottingham University Hospitals, Nottingham, UK; ^2^ Section of Epidemiology and Biostatistics, Leeds Institute of Cancer and Pathology, University of Leeds; Leeds, UK; ^3^ NIH Chemical Genomics Center, National Center for Advancing Translational Sciences, National Institutes of Health, 9800 Medical Center Drive, Rockville, Maryland 20850, USA; ^4^ Academic Haematology, Division of Oncology, School of Medicine, University of Nottingham, Nottingham University Hospitals, Nottingham, UK; ^5^ Cancer Biology Unit, Division of Oncology, School of Medicine, University of Nottingham, Nottingham University Hospitals, Nottingham, UK; ^6^ School of Science and Technology, Nottingham Trent University, Clifton campus Nottingham, UK; ^7^ Laboratory of Molecular Gerontology, Biomedical Research Center, National Institute on Aging, National Institutes of Health, Baltimore, Maryland 21224-6825, USA

**Keywords:** PTEN, DNA repair, APE1, APE1 inhibitors, synthetic lethality

## Abstract

Phosphatase and tensin homolog (*PTEN*) loss is associated with genomic instability. *APE1* is a key player in DNA base excision repair (BER) and an emerging drug target in cancer. We have developed small molecule inhibitors against *APE1* repair nuclease activity. In the current study we explored a synthetic lethal relationship between *PTEN* and *APE1* in melanoma. Clinicopathological significance of *PTEN* mRNA and *APE1* mRNA expression was investigated in 191 human melanomas. Preclinically, *PTEN*-deficient *BRAF*-mutated (UACC62, HT144, and SKMel28), *PTEN*-proficient *BRAF*-wildtype (MeWo), and doxycycline-inducible *PTEN*-knockout *BRAF*-wildtype MeWo melanoma cells were DNA repair expression profiled and investigated for synthetic lethality using a panel of four prototypical *APE1* inhibitors. In human tumours, low *PTEN* mRNA and high *APE1* mRNA was significantly associated with reduced relapse free and overall survival. Pre-clinically, compared to *PTEN*-proficient cells, *PTEN*-deficient cells displayed impaired expression of genes involved in DNA double strand break (DSB) repair. Synthetic lethality in *PTEN*-deficient cells was evidenced by increased sensitivity, accumulation of DSBs and induction of apoptosis following treatment with *APE1* inhibitors. We conclude that *PTEN* deficiency is not only a promising biomarker in melanoma, but can also be targeted by a synthetic lethality strategy using inhibitors of BER, such as those targeting *APE1*.

## INTRODUCTION

Base excision repair (BER) is a critical and highly conserved mechanism for the repair of damage induced by alkylation and oxidation of DNA, including by chemotherapy and ionising radiation [1]. Abasic sites (also known as apurinic/apyrimidinic or AP sites) are cytotoxic obligate repair intermediates generated during BER and processed by human apurinic/apyrimidinic endonuclease 1 (*APE1*). *APE1* cleaves the phosphodiester DNA backbone 5' to the AP site prior to further processing via either the short patch or the long patch BER pathway. Unrepaired AP sites generate single strand breaks, which stall replication fork progression and induce DNA double strand breaks (DSBs) that are toxic to the cell at high density [2].

*APE1* is a multifunctional protein [1, 3]. In addition to BER functions, it possesses N-terminus redox activity, which can activate pro-angiogenic and pro-survival transcription factors. *APE1* also has roles in acetylation-mediated gene regulation and RNA quality control [4]. SiRNA-mediated *APE1* downregulation induces AP site accumulation and is associated with hypersensitivity to DNA damaging agents, including alkylators and ionising radiation [1]. Overexpression of *APE1* confers resistance to these agents, both *in vitro* and *in vivo* [1]. Furthermore, exposure to alkylating agents causes upregulation of endogenous *APE1* levels, suggesting a role in the development of treatment resistance [5]. *APE1* expression in human tumours may have prognostic or predictive significance in patients [1].

In light of the evidence presented above, *APE1* is an emerging anti-cancer drug target. [1, 3]. We have initiated drug development programmes to identify novel inhibitors of *APE1* DNA repair function [6-11]. Several of these compounds have shown promising preclinical activity, including the potentiation of the cytotoxicity of the alkylating agent temozolomide in cancer cell lines. More recently, we have demonstrated synthetic lethality of *APE1* inhibition in BRCA-deficient cell systems [12], analagous to results observed with PARP inhibitors currently under development for treatment of HR-deficient cancer [13, 14].

Phosphatase and tensin homolog (*PTEN*) is a negative regulator of the anti-apoptotic PI3K/Akt pathway [15]. *PTEN* mutation is reported in 5-20% of primary melanomas, although *PTEN* mutation is more frequently seen in melanoma cell lines (30-50%) [16, 17]. Furthermore, transcriptional and translational repression of *PTEN* function has been reported in up to 65% of melanomas [18]. In addition to its inositol phosphatase function, *PTEN* has recently been implicated in the maintenance of genomic integrity [19-21]. *PTEN*-null cells are associated with centromere-clustered chromosome breakages, possibly due to interaction with the centromeric protein CENP-C, which is vital for centromeric stability during mitosis. Additionally, *PTEN* might function as a transcriptional regulator of the critical homologous recombination (HR) protein *RAD51* via the transcription factor Egr-1 [19-21]. Alternatively, *PTEN* loss may be associated with altered expression of the *RAD51* paralogs [22] or impaired HR factor recruitment to DNA damage due to cell cycle checkpoint defects [20]. SUMOylation may be essential for *PTEN* DNA repair functions by directing nuclear *PTEN* localisation, with *PTEN*-null or non-SUMOylatable mutant cells exhibiting enhanced sensitivity to DNA damaging agents [23]. In keeping with these findings, *PTEN* −/− cells have been demonstrated to possess a HR defect that is associated with synthetic lethality following PARP inhibitor exposure [24]. However, although an association between *RAD51* deficiency, impaired HR and *PTEN* deficiency has been demonstrated in colorectal cancer cells [24] and endometrial cancer cells [25], the association was not demonstrated in prostate cancer models [22].

Loss of *PTEN* may promote melanoma development [26], possibly as a cooperating mutation with *BRAF V600E* [27]. Oncogenic *BRAF* V600 driver mutations have recently emerged as a key therapeutic target [28], leading to the development of vemurafanib [29]. Despite *BRAF*-targeted therapy, many patients eventually progress and succumb to the disease. Interestingly, a recent study has suggested that *PTEN* loss may contribute to *BRAF* inhibitor resistance in melanoma [30]. Therefore, development of therapeutic strategies targeting *PTEN* deficiency is highly desirable. In the current study, we hypothesised a synthetic lethal relationship between *PTEN* and *APE1* in melanoma. We have measured mRNA expression of *PTEN* and *APE1* in 191 human melanomas and correlated this with clinical and pathological factors. We have confirmed the utility of *APE1* inhibitors in the presence of *PTEN* deficiency in melanoma cell lines.

## RESULTS

### Prognostic significance of *PTEN* mRNA and *APE1* mRNA expression in human melanomas

**Figure 1:**
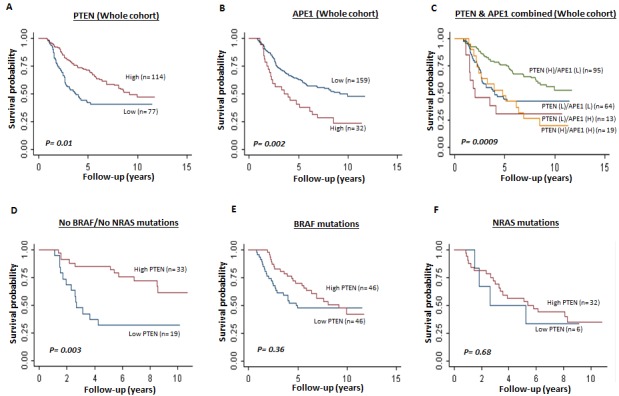
Kaplan Meier curves showing overall survival in melanoma A. Whole cohort (PTEN mRNA high and low, *p=0.01*). B. Whole cohort (APE1 mRNA high and low, *p=0.002*). C. Whole cohort (PTEN and APE1 m RNA combined high and low, *p=0.0009*). D. Tumours with no BRAF/NRAS mutations (PTEN mRNA high and low, *p=0.003*). E. Tumours with BRAF V600E mutation (PTEN mRNA high and low, *p=0.36*). F. Tumours with NRAS mutations (PTEN mRNA high and low, *p=0.68*).

Patient demographics of the 191 cases are summarized in Supplementary Table S1. The clinicopathological association data are summarised in Supplementary Table S2. Relapse free and overall survival data are summarized in Supplementary Table S3. Low *PTEN* and high *APE1* mRNA expression associated with presence of vascular invasion (p=0.05) and high mitotic rate (p=0.4), respectively. In the whole cohort (n=191), low *PTEN* mRNA expression was significantly associated with poor relapse free survival and overall survival (Supplementary Table S3 and Figure 1A). High *APE1* mRNA expression was also significantly associated with poor relapse free survival and overall survival (supplementary Table S3 and Figure 1B) in the whole cohort. When *PTEN* and *APE1* are considered together, patients with tumours that exhibit high *PTEN* and low *APE1* mRNA expression have a significantly better prognosis compared to tumours that have low *PTEN*/high *APE1* mRNA expression or low *PTEN*/low *APE1* mRNA expression or high *PTEN*/high *APE1* mRNA expression (Figure 1C).

Given the evidence in murine models that low *PTEN* and *BRAF* V600 mutations results in development of metastatic melanoma [27], we conducted an exploratory analysis based on *PTEN* mRNA expression and *BRAF*/NRAS status. None of the patients with *BRAF* V600 mutation had received vemurafanib (*BRAF* inhibitor) therapy. In tumours that had no *BRAF* or *NRAS* mutation, low *PTEN* was significantly associated with poor overall survival [*HR 95%CI)=0.27 (0.12, 0.64), P=0.003*] (Figure 1D). In tumours that have *BRAF* mutation (Figure 1B) [*HR (95%CI)=0.81 (0.46, 1.43), p=0.47*] and in *NRAS* mutants, low *PTEN* level did not significantly influence prognosis (Figure 1C) [*HR 95%CI)=0.88 (0.29, 2.69), p=0.82*]. Taken together, the data suggest that *PTEN* deficiency is a promising prognostic marker in *BRAF* wild type melanomas.

### *PTEN*-deficient melanoma cell lines exhibit altered DSB repair protein levels

**Figure 2:**
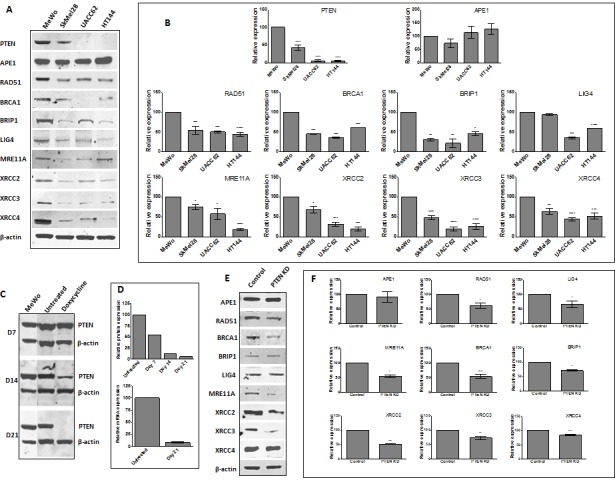
**A.** Representative Western blots of PTEN and selected DNA repair proteins in human melanoma cell lines. B. Quantification of PTEN and selected DNA repair factor protein levels in melanoma cell lines. C. Generation of doxycycline-inducible PTEN knockdown MeWo cells. Following lentiviral transduction, continuous doxycycline exposure induced PTEN shRNA expression. Whole cell lysates were examined for PTEN protein levels every 7 days, with almost complete absence observed by day 21. This was confirmed by mRNA analysis by qRT-PCR (D). E. Representative Western blots and quantification (F) of PTEN and selected DNA repair proteins in control and PTEN knockdown (KD) cell line. * p<0.05, ** p<0.01, *** p<0.001, **** p<0.0001 compared to control cells.

The emerging role of *PTEN* in DNA repair [23] and the association between low *PTEN* and impaired DNA DSB repair [19-21] implies that *PTEN*- deficient melanoma cells with defective DSB repair may be more reliant upon BER to maintain genomic stability. To investigate the hypothesis that *PTEN* loss is associated with impaired HR, DSB repair protein levels were examined in melanoma cell lines. MeWo (*BRAF* wildtype, NRAS wildtype), SkMel28 (*BRAF V600E* mutant, NRAS wildtype), HT144 (*BRAF V600E* mutant, NRAS wildtype) and UACC62 (*BRAF V600E* mutant, NRAS wildtype) melanoma cell lines were screened for *PTEN* protein. As shown in Figure 2A and 2B, western blot analysis confirmed high *PTEN* protein levels in MeWo cells. UACC62 and HT144 cells demonstrate an almost complete absence of *PTEN* protein; SkMel28 exhibits an intermediate level. All four cell lines are proficient in *APE1* expression. In keeping with previous studies [21, 24], *PTEN*-deficient HT144 and UACC62 melanoma cells were also *RAD51*-deficient. *RAD51* level was also lower in SkMel28 cells compared to MeWo. However, Fraser et al. did not observe *RAD51* deficiency in *PTEN*-deficient prostate cancer cells [22]. Therefore, for additional clarification, we explored protein levels of other key DSB repair factors. As shown in Figure 2A and 2B, *PTEN*-deficient cells exhibited consistently lower levels of a number of DSB repair proteins compared to *PTEN*-proficient MeWo.

We proceeded to generate a doxycycline inducible *PTEN*-knockdown, *BRAF*-proficient MeWo cell line. Figure 2C and 2D demonstrates time-dependent knockdown of *PTEN* protein expression following doxycycline induction of lentiviral shRNA transduced into MeWo cells. By day 7, *PTEN* protein level was reduced to ~50% baseline, reaching >90% by day 14. We confirmed reduced *PTEN* transcript production at day 21 by qRT-PCR (Figure 2D). Western blot examination confirmed that levels of a number of DSB repair proteins were reduced in *PTEN*-knockdown cells compared to non-induced controls (Figure 2E and 2F). Taken together, these data confirm that *PTEN* loss is associated with reduced DSB repair protein levels in melanoma cells.

### *PTEN*-deficient melanoma cells have dysregulated DNA repair mRNA expression

To investigate DNA repair expression at the mRNA level, we profiled a panel of 88 DNA repair genes in MeWo, *PTEN*-knockdown MeWo, SkMel28, HT144 and UACC62 melanoma cells using the RT2 Profiler DNA Repair PCR array. Significantly reduced mRNA expression of several HR repair genes was observed in *PTEN*-deficient cells (summarised in Supplementary Table S5 and Supplementary Figure S1). This included several repair factors that have previously been implicated in *PTEN* deficiency, including *RAD51* [21, 24, 37], *MRE11* [22], and the *RAD51* paralogs *RAD51B*, *RAD51C*, *RAD51*D, *XRCC2* and *XRCC3* [38]. We also observed low expression of a number of genes involved in non-homologous end joining(NHEJ), as well as nucleotide excision repair(NER) and mismatch repair(MMR). Interestingly, significantly increased mRNA expression of ATM, CCNH and DDB2 was also observed. Taken together, the data suggest that *PTEN*-deficient melanoma cells have complex patterns of DNA repair dysregulation with consistent downregulation of genes involved in DSB repair.

### *PTEN*-deficient melanoma cells are sensitive to *APE1* inhibitors

**Figure 3:**
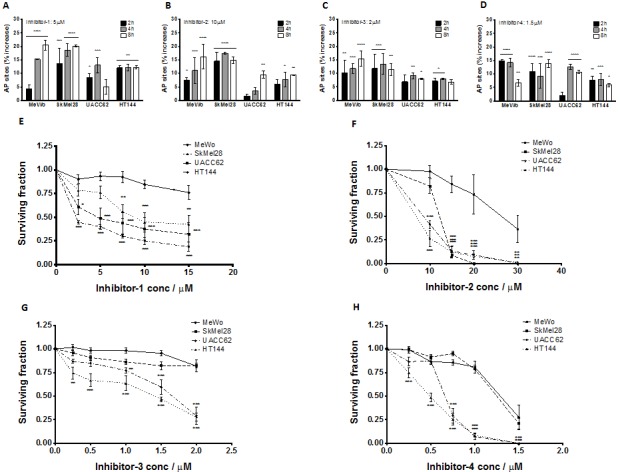
Aldehyde reactive probe assay confirming target inhibition in MeWo, SkMel28, UACC62 and HT144 cells treated with inhibitor -1 (A), inhibitor -2 (B), inhibitor -3 (C) and inhibitor -4 (D) Clonogenic survival assays in MeWo, SkMel28, UACC62 and HT144 cells treated with inhibitor-1 (E), inhibitor-2 (F), inhibitor-3 (G) and inhibitor-4 (H). Inhibitors were added at the indicated concentrations (see methods for details). * p<0.05, ** p<0.01, *** p<0.001, **** p<0.0001 compared to MeWo (*PTEN*-wildtype).

**Figure 4:**
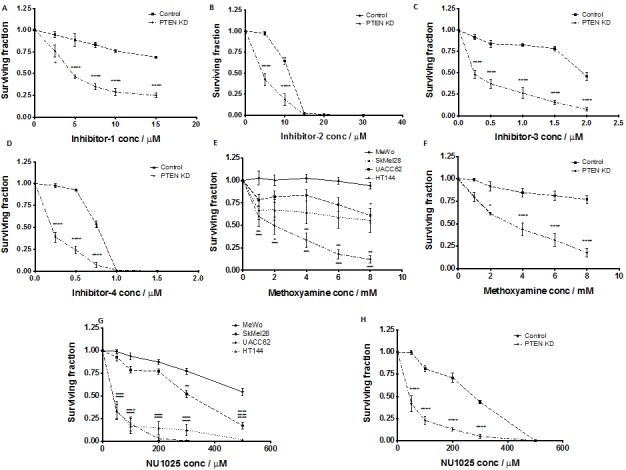
Clonogenic survival assays in control MeWo cells and PTEN knockdown (KD) MeWo cells treated with inhibitor-1 (A), inhibitor-2 (B), inhibitor-3 (C) and inhibitor-4 (D) Clonogenic survival assays in MeWo, SkMel28, UACC62 and HT144 cells treated with methoxyamine (E). Clonogenic survival assays in control MeWo cells and PTEN knockdown (KD) MeWo cells treated with methoxyamine (F). Clonogenic survival assays in MeWo, SkMel28, UACC62 and HT144 cells treated with NU1025 (G). Clonogenic survival assays in control MeWo cells and PTEN knockdown (KD) MeWo cells treated with NU1025 (H). Inhibitors were added at the indicated concentrations (see methods for details). * p<0.05, ** p<0.01, *** p<0.001, **** p<0.0001 compared to MeWo (PTEN-wildtype).

As *PTEN*-deficient cells exhibited differential expression of HR factors, blockade of BER through *APE1* inhibition could lead to synthetic lethality. To test this hypothesis, we conducted studies in clinically relevant *PTEN*-deficient and *PTEN*-proficient melanoma cells using a panel of *APE1* inhibitors. Four prototypical *APE1* inhibitors were evaluated (chemical structures are shown in Supplementary Figure S2) in MeWo, SKMel28, UACC62, HT144 and *PTEN*-knockdown MeWo cells. We first confirmed target inhibition** using the ARP assay. A significant accumulation of AP sites in genomic DNA was demonstrated following 2, 4 and 8 hours exposure to inhibitor 1, inhibitor 2, inhibitor 3 or inhibitor 4 (Figures 3A-D). We then evaluated whether these inhibitors could induce selective cytotoxicity in *PTEN*-deficient cells using clonogenic survival assays. Treatment with each inhibitor resulted in reduced survival of *PTEN*-deficient SKMel28, UACC62, and HT144 cells in comparison to *PTEN*-proficient MeWo cells (Figure 3E-3H). Similarly, *PTEN*-knockdown MeWo cells exhibited significantly increased sensitivity to *APE1* inhibitor treatment compared to control cells (Figures 4A-4D).

**Figure 5:**
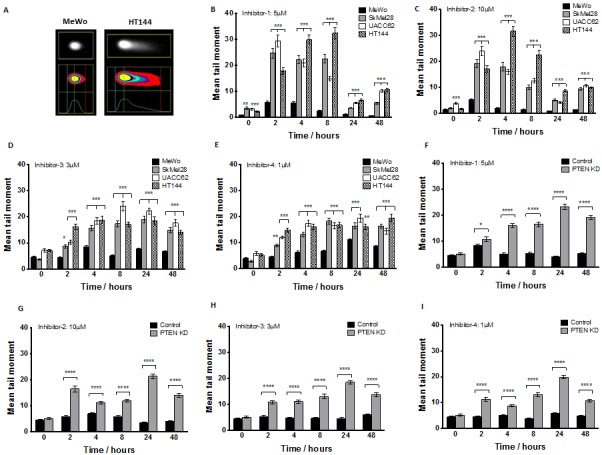
Neutral comet assay was performed at various time points after APE1 inhibitor exposure as indicated in methods A. Example of increased DNA damage comet ‘tail’ in PTEN-deficient HT144 compared to MeWo cells following 24 hours exposure to inhibitor-1. Significantly increased mean tail moment was observed in PTEN-deficient SkMel28, UACC62 and HT144 cells compared to MeWo cells following treatment with inhibitor-1 (B), inhibitor-2 (C), inhibitor 3 (D) and inhibitor 4 (E). Similarly, significantly increased mean tail moment was observed in PTEN knockdown (KD) MeWo cells compared to control MeWo cells treated with inhibitor-1 (F), inhibitor-2 (G), inhibitor 3 (H) and inhibitor 4 (I). ** p<0.01, *** p<0.001, compared to MeWo (PTEN-wildtype).

To confirm that selective toxicity is due to *APE1* inhibtion, we utilised the indirect *APE1* inhibitor methoxyamine. Methoxyamine binds irreversibly to AP sites in DNA [36], preventing *APE1* from processing the adducted AP lesions. Figure 5E demonstrates that methoxyamine is more lethal to *PTEN*-deficient SKMel28, UACC62, and HT144 cells than to *PTEN*-proficient MeWo cells. Similarly, *PTEN*-knockdown MeWo cells were more sensitive to methoxyamine compared to *PTEN*-proficient control cells (Figure 4F). To further establish whether this selective cytotoxicity was due to obstruction of BER, we investigated NU1025, a PARP inhibitor that blocks the BER-related single strand break repair (SSBR) pathway [39]. Figure 4G demonstrates the increased toxicity of NU1025 in *PTEN*-deficient SKMel28, UACC62 and HT144 cells compared to *PTEN*-proficient MeWo cells. This result was repeated in *PTEN*-knockdown MeWo cells, which were more sensitive to NU1025 (Figure 4H) than *PTEN*-proficient MeWo cells. The data is consistent with a previous study in which *PTEN*-deficient colorectal cells were found to exhibit increased sensitivity to PARP inhibition [24].

### Selective sensitivity of *PTEN*-deficient melanoma cells following *APE1* inhibition results from increased DNA damage accumulation

The clonogenic survival data provides compelling evidence for selective toxicity of *APE1* inhibitors in *PTEN*-deficient melanoma cells. To provide mechanistic evidence that *APE1* inhibition leads to synthetic lethality in *PTEN*-deficient cells, we investigated the functional consequence of *APE1* inhibitor treatment. The neutral COMET assay detects single and double strand breaks(DSBs) in DNA. Figures 5A-5I summarise the results for MeWo, SKMel28, UACC62, HT144 and *PTEN*-knockdown MeWo cells treated with *APE1* inhibitors. Mean tail moment was increased in all samples after *APE1* inhibitor exposure compared to pre-treatment samples, and was significantly higher in *PTEN*-deficient cells at 2, 4, and 8 hours in comparison to *PTEN*-proficient cells, with damage persisting to 48 hours. The data demonstrates that *PTEN*-deficient cells accumulate greater numbers of DNA breaks after exposure to an *APE1* inhibitor compared to *PTEN*-proficient cells.

**Figure 6:**
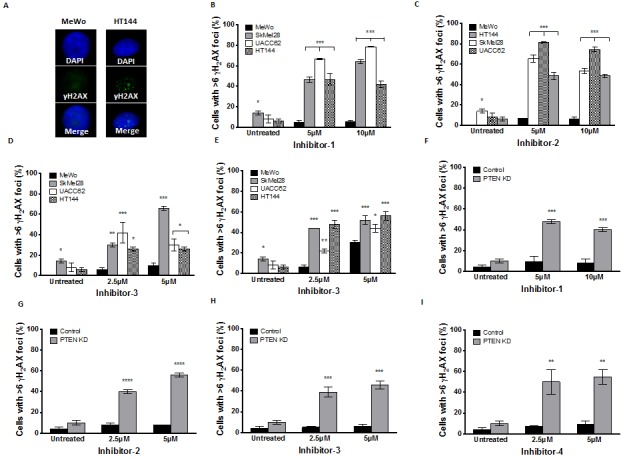
**A.** γH2AX immunocytochemistry following 24 hours inhibitor-1 exposure demonstrates increased foci in PTEN-deficient HT144 cells compared to PTEN-wildtype MeWo. Significantly increased γH2AX foci was observed in PTEN-deficient SkMel28, UACC62 and HT144 cells compared to MeWo cells following 24 hours treatment with inhibitor-1 (B), inhibitor-2 (C), inhibitor-3 (D) or inhibitor-4 (E). Similar significantly increased γH2AX foci was observed in PTEN knockdown (KD) MeWo cells compared to control MeWo cells following 24 hours treatment with inhibitor-1 (F), inhibitor-2 (G), inhibitor-3 (H) or inhibitor-4 (I). * p<0.05, ** p<0.01, *** p<0.001, compared to MeWo (PTEN-wildtype).

Phosphorylation of H2AX at serine 139 (γH2AX) is induced by DSBs, and can be used as a marker of DSB formation. Following inhibitor exposure, γH2AX immunocytochemistry was performed in MeWo, SKMel28, UACC62, HT144 and *PTEN*-knockdown MeWo cells. As shown in Figures 6A-6I, the percentage of cells with more than six γH2AX foci following inhibitor exposure was significantly higher in *PTEN*-deficient cells compared to *PTEN*-proficient control cells. These data provide additional evidence that *PTEN*-deficient cells accumulate DSBs at an increased rate after exposure to an *APE1* inhibitor relative to *PTEN*-proficient MeWo cells.

**Figure 7:**
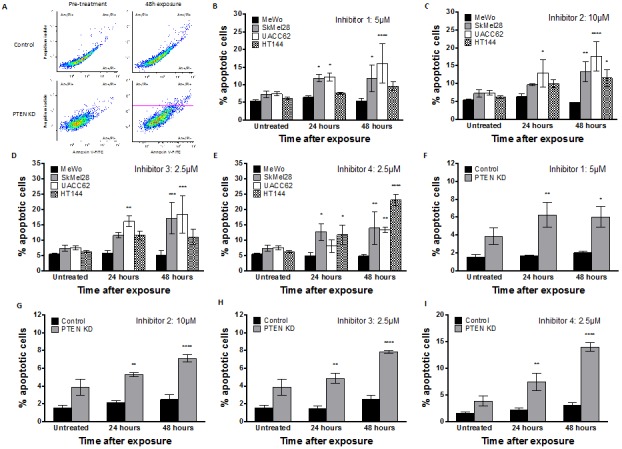
**A.** Apoptosis detection by FITC-annexin V flow cytometric analysis demonstrates an increased apoptotic fraction in HT144 following 48 hours exposure to inhibitor-1, as observed by increased FITC-Annexin V staining in upper and lower right quadrants. Significantly increased apoptotic fraction was observed in PTEN-deficient SkMel28, UACC62 and HT144 cells compared to MeWo cells following 48 hours treatment with indicated concentrations of inhibitor-1 (B), inhibitor-2 (C), inhibitor-3 (D) or inhibitor-4 (E). Similar significantly increased apoptotic fraction was observed in PTEN knockdown (KD) MeWo cells compared to control MeWo cells following 48 hours treatment with inhibitor-1 (F), inhibitor-2 (G), inhibitor-3 (H) or inhibitor-4 (I). * p<0.05, ** p<0.01, *** p<0.001, compared to MeWo (PTEN-wildtype).

DSB accumulation activates a complex cell cycle checkpoint response that may result in eventual induction of apoptosis. Apoptosis detection by FITC-annexin V flow cytometric analysis was therefore performed in MeWo, SKMel28, UACC62, HT144 and *PTEN*-knockdown MeWo cells following exposure to inhibitor 1, inhibitor 2, inhibitor 3, or inhibitor 4 for 24 or 48 hours. As shown in Figure 7A-7I, the percentage of cells undergoing apoptosis following *APE1* inhibitor exposure was significantly higher in *PTEN*-deficient cells in comparison to *PTEN*-proficient MeWo cells.

Taken together, these functional studies provide compelling evidence that *APE1* inhibition induces a synthetic lethal response in *PTEN*-deficient cells by causing accumulation of abasic sites and subsequent strand breaks, and ultimately the induction of apoptosis.

## DISCUSSION

*PTEN* is a key regulator of the anti-apoptotic PI3K/Akt pathway [15], and emerging evidence suggests a role in DNA repair [19-21]. The role of *PTEN* in the regulation of *RAD51* remains controversial [24]. However, a number of recent studies provide compelling evidence that nuclear *PTEN* may have essential roles in DNA repair [40, 41], and that *PTEN* deficiency may be targeted in a synthetic lethality approach by inhibitors of SSBR [24]. Our primary hypothesis is that this synthetic lethality approach may be applied to *PTEN*-deficient melanomas using BER inhibitors such as those targeting *APE1*. We initially investigated the clinicopathological significance of *PTEN* mRNA and *APE1* mRNA in melanoma. Low *PTEN* was associated with poor survival overall but the effect was absent in *BRAF* and *NRAS-*mutated tumours, and only statistically significant in tumours without *BRAF* or *NRAS* mutations. This may be related to the complex tumour-suppressing function of *PTEN*, including its roles as a negative regulator of the anti-apoptotic PI3K/Akt pathway and in DNA repair regulation. That the influence of *PTEN* expression on prognosis was more pronounced in tumours without *BRAF* or NRAS mutations is a new observation, and suggests that *PTEN* loss may be a key biomarker in *BRAF*-wildtype tumours. Taken together, the clinical data are consistent with pre-clinical study that implicates *PTEN* loss in melanoma progression [26]; however, the relatively small patient numbers in the various subgroups is a limitation of our study. Our finding that high *APE1* mRNA is associated with poor survival in melanoma is supported by previous studies [42] and provides additional evidence for targeting *APE1* in melanoma.

The success of PARP inhibitors in BRCA-deficient breast and ovarian tumours [13, 14] implies that a synthetic lethality approach may be an attractive strategy in melanomas with DSB repair deficiency. *PTEN*-deficient melanoma cells with defective DSB repair may be reliant upon BER as a frontline defence to prevent damage accumulation, replication fork collapse, DSB formation and consequent cell death. Thus, blockade of BER through *APE1* inhibition could lead to synthetic lethality in *PTEN*-deficient cells. To test this hypothesis, we first profiled *PTEN*-deficient and PTEN-proficient melanoma cells for DNA repair factor expression, finding that expression of several genes involved in HR was impaired at protein and mRNA level in *PTEN*-deficient cells. In addition we observed downregulation of genes involved in NHEJ, NER, MMR and MGMT. Given the role of *PTEN* in the regulation of HR expression as well as in HR recruitment to sites of DNA damage [19-21], it is perhaps not surprising that genomic instability in *PTEN*-null cells may, over a period of time, eventually lead to acquisition of new defects in other DNA repair pathways. Recent studies that positively link *PTEN* with NER [43], MMR [44], and MGMT expression [45] suggests that such a mechanism may be operating in cells, but detailed mechanistic studies are required to confirm this hypothesis. An interesting observation across *PTEN*-deficient cell lines was the loss of BRCA1 protein expression, consistent with previous evidence in UACC62 melanoma cells (http://cancer.sanger.ac.uk/cancergenome/projects/cosmic/). This observation is also consistent with a recent study in breast cancer, wherein high frequency of *PTEN* loss was observed in BRCA1-associated breast tumours [46]. In a separate study, *PTEN* loss was found to be a predictor of BRCA1 germ-line mutations in women with early onset breast cancer [47]. Taken together, this evidence suggests a possible functional link between *PTEN* and BRCA1 in melanomas. These observations, however, require detailed mechanistic studies to confirm functional interactions between *PTEN* and BRCA1.

*PTEN*-deficient melanoma cells were shown in the current study to be sensitive to *APE1* inhibitors. Similar hypersensitivity observed following exposure to the PARP inhibitor NU1025 is consistent with previous observations using *PTEN*-deficient colorectal cells [24]. We have concluded that the interrelationship between BER and *PTEN* may be a valid therapeutic target in melanoma for the following reasons: a) *PTEN*-deficient cells are sensitive to *APE1* repair domain inhibitors; b) *PTEN*-deficient cells are sensitive to methoxyamine, an indirect inhibitor of *APE1*; c) *PTEN*-deficient cells are sensitive to the PARP inhibitor NU1025; and d) upon *APE1* inhibitor treatment, *PTEN*-deficient cells accumulate DNA DSBs, resulting in the induction of apoptosis. It should be noted that the observed level of apoptosis in *PTEN*-deficient cells following inhibitor exposure is lower than may be predicted from survival analyses using clonogenic assays. Although apoptotic cell accumulation may occur over a period of time following chronic exposure to *APE1* inhibitor, another possible explanation is ‘synthetic sickness’, a synthetic lethal-type relationship wherein the overall outcome is reduced fitness rather than cellular lethality [48]. Accumulation of DNA damage, including specific DSB accumulation, has been associated with the induction of cellular senescence [49]. More recently, *PTEN* loss has also been shown to induce a senescent phenotype [50]. Therefore we speculate that DSB accumulation in *PTEN*-deficient cells following *APE1* inhibition, besides induction of apoptosis, may also drive the cell population into senescence, contributing to reduced clonogenicity. Although further study is required to assess for definitive evidence of senescence induction in this setting, our data raise the intriguing possibility of a future avenue for pro-senescence therapy.

In conclusion, our study provides the first evidence that blockage of BER by *APE1* inhibition has attractive potential for a novel therapeutic approach in *PTEN*-deficient melanomas. This strategy could have significant translational applications for personalised therapy in melanoma patients.

## METHODS

### Clinical study

Gene expression analysis: Whole genome gene expression (~30,000 probes) was measured using Illumina DASL approach in 240 formalin fixed primary melanoma tumours from Leeds Melanoma Cohort [31], including 29 duplicates to serve in QC checks. After QC, the cohort contained 191 patients including 106 who had relapsed and 101 who had died. Histology data were derived from clinical histopathology reports. For a subset of tumors diagnostic H+E slides were reviewed by a dermatopathologist (Dr Andy Boon (Cohort study) to standardize reporting across the specimens. *BRAF* and NRAS mutation status was also available for 182 of these tumours, derived using pyrosequencing as previously described [32]. The study was approved by the National Research ethics committee (UK).

### Statistical analyses

Data was normalised using background correction and robust spline smoothing with Lumi R package. The associations between *PTEN* and *APE1* mRNA expressions and histological factors were assessed using linear and logistic regression of log_2_ transformed gene expression data. Separate and joint effects of *PTEN* and *APE1* mRNA expressions on relapse-free and overall survival was tested. Expression data were used as continuous after log2 transformation and after dichotomisation using expression cut-offs determined using X-Tile software [33]. In stratified analysis, Cox proportional hazard model was applied on dichotomized *PTEN* expression stratifying on presence of *BRAF* mutations, NRAS mutations and no mutations.

### Cell lines

Clinically relevant previously well-characterised melanoma cell lines were chosen for the pre-clinical study. MeWo is a *BRAF* wildtype and NRAS wildtype melanoma cell line. SkMel28, HT144 and UACC62 are *BRAF* V600 mutant and NRAS wildtype melanoma cells lines (http://cancer.sanger.ac.uk/cancergenome/projects/cosmic/).

### Generation of doxycycline inducible *PTEN* knockdown MeWo cell line by lentiviral shRNA transduction

TRIPZ shRNA plasmid against *PTEN* was purchased from Thermo Scientific (Clone ID: V3THS_312158: 5'-GGAAAGAATCAAGGAGG-3', Loughborough, UK). Lentiviral production was performed per the supplied protocol using the Lenti-X high-titer lentiviral packaging system in Lenti-X 293T cells (Clontech, Mountain View, USA). After 48 hours incubation, lentivirus-containing supernatant was harvested and applied to MeWo cells at an MOI of 1 or 3. Once stable in culture, puromycin selection was initiated at a concentration of 1μg/ml. To induce shRNA expression, doxycycline was added to culture medium at a concentration of 0.5-1μg/ml and replaced every 72 hours. Confirmation of reduced transcript production by qRT-PCR was performed on an Applied Biosystems 7500 FAST cycler using Qiagen PCR primers against *PTEN* [Qiagen Quantitect primers (HS_*PTEN*_4_SG and HS_GAPDH_1_SG)]. Absence of *PTEN* protein expression was confirmed on Western blot.

### qRT-PCR analysis of DNA repair gene expression in melanoma cell lines

RNA was extracted from melanoma cell lines using the RNeasy Mini Kit (Qiagen) and quantified using a microvolume spectrophotometer. cDNA synthesis was performed using the RT2 First Strand Kit (Qiagen). Real time PCR was carried out on an Applied Biosystems 75000 FAST cycler in a commercially available 96-well plate format, allowing assessment of 88 DNA repair genes simultaneously (RT2 Profiler DNA Repair PCR Array). Threshold cycle was calculated for each well and exported to online software for further analysis (www.SABiosciences.com/pcrarraydataanalysis.php). All experiments were performed in triplicate.

### Western blot analysis

Primary antibody details are summarised in Supplementary Table S4. Protein expression was examined by infrared dye-labelled secondary antibody (Li-Cor, 1:15000 dilution) detected by Li-Cor Odyssey Scanner. All experiments were performed in triplicate.

### *APE1* inhibitors and other compounds

We have previously identified a number of *APE1* inhibitors. [34, 35] Cell biology experiments performed here utilised structural analogues of *N*-(4-fluorophenyl)-2-[4-phenylsulfonyl-2-(p-tolyl)oxazol-5-yl]sulfanyl-acetamide (previously characterised in [12]: N-(4-fluorophenyl) -2- (2-phenyl-4- phenylsulfonyl-1,3-oxazol-5-yl]sulfanyl-acetamide (Inhibitor-1) and N-(4-fluorophenyl)-2-(2-phenyl-4-phenylsulfonyl-oxazol-5-yl)sulfanyl-acetamide (Inhibitor-2), purchased from ChemDiv. Additional inhibitors previously identified by Rai *et al.* [35] were synthesised by the National Institutes of Health (NIH) Chemical Genomics Center (NCGC): *N*-(3-(benzo[d]-thiazol-2-yl)-6-isopropyl-4,5,6,7-tetrahydrothieno[2,3-c]-pyridin-2-yl)acetamide (Inhibitor-3) and its analogue *N*-(3-(benzo[*d*]thiazol-2-yl)-5,6-dihydro-4*H*-thieno[2,3-*c*]pyrrol-2-yl)acetamide (Inhibitor-4). *APE1* inhibitors investigated here are highly potent and specific for *APE1*. They do not bind to DNA and have no activity against *E. coli* endonuclease IV (a functional homolog with no sequence or structural homology to *APE1*). IC_50_ for *APE1* endonuclease activity inhibiton in purified protein fluoresence based biochemical assays are as follows; inhibitor 1= 0.2µM, inhibitor 2= 0.1µM, inhibitor 3= 2µM and inhibitor 4=3.3µM. The compounds also block AP site cleavage activity in HeLa whole cell extract assays, and potentiate the cytotoxicity of alkylating agents in cancer cell lines [34, 35]. Molecular modelling studies indicate that these *APE1* inhibitors dock onto the active site of *APE1* [8, 34]. Methoxyamine, a non-specific indirect inhibitor of *APE1*, binds irreversibly to AP sites in DNA [36] and prevents *APE1* (and endonuclease IV) from processing the adducted AP lesion. Methoxyamine (indirect *APE1* inhibitor) was purchased from Sigma. NU1025 (PARP inhibitor) was purchased from Tocris Bioscience.

### Clonogenic survival assay

Cell lines were plated into 6-well plates at a density of 200-400 cells per well. Cells were allowed to adhere for 4 hours, after which inhibitory compound was added at varying concentrations. Cells were incubated for 14 days under normal incubator conditions. After this time, media was discarded and cells were stained using 10% crystal violet in 70% aqueous ethanol to allow quantification of colony number. All experiments were performed in triplicate.

### Aldehyde Reactive Probe (ARP) assay

Cell-based ARP assay was performed according to the manufacturer's instructions (Abcam). Cells were plated at high density in a 96-well plate, allowed to grow for 24 hours, then treated with inhibitor. Results were presented as percent increase in fluorescence as a surrogate for AP site accumulation.

### Neutral COMET assay

Cell lines were plated into 6cm petridishes at a density of 10^5^ cells per plate. Cells were allowed to adhere for 24 hours, after which time inhibitory compound was added at a single concentration. Cells were harvested at 0, 2, 4, 8, 24 and 48 hour time points, and neutral COMET assay was performed as described previously [12]. All experiments were performed in triplicate.

### γH2AX immunocytochemistry

Cell lines were seeded onto sterile coverslips in 6-well plates at a density of 10^5^ cells per well. Cells were allowed to adhere for 24 hours, after which time inhibitory compound was added. After 24 hours exposure, γH2AX immunocytochemistry was performed [12]. The number of γH2AX foci per nucleus was determined in 100 cells per slide. Nuclei containing more than 6 γH2AX foci were considered positive. All experiments were performed in triplicate.

### Apoptosis detection by FITC-annexin V flow cytometric analysis

Cell lines were plated into 6-well plates at a density of 5 × 10^5^ cells per well. Cells were allowed to adhere for 24 hours, after which time inhibitory compound was added. After 24 hours exposure, cells were gently trypsinised, washed twice in ice-cold PBS, and resuspended in Annexin V binding buffer (FITC Annexin V Apoptosis Detection Kit I, BD Pharmingen). Cells were incubated for 15 minutes in the dark with FITC-Annexin V and propidium iodide (PI), then analysed by flow cytometry using a BC Accuri C6 Flow Cytometer. The percentage of induced apoptosis (FITC-Annexin V positive, PI negative and FITC-Annexin V positive, PI positive) was determined by comparison to a control population of untreated cells. All experiments were performed in triplicate [12].

## SUPPLEMENTARY FIGURES AND TABLES


