# Supplementary material for: Targeting human apurinic/apyrimidinic endonuclease 1 (APE1) in phosphatase and tensin homolog (PTEN) deficient melanoma cells for personalized therapy
Source: Oncotarget. 2014 Apr 27;5(10):3273–86. doi: 10.18632/oncotarget.1926 (PMC4102809; doi:10.18632/oncotarget.1926)
Supplement: Supplementary file 1 [file oncotarget-05-3273-s001.pdf]

Targeting human apurinic/apyrimidinic endonuclease 1 (APE1) in phosphatase and tensin homolog (PTEN) deficient melanoma cells for personalized therapy

Supplementary Material

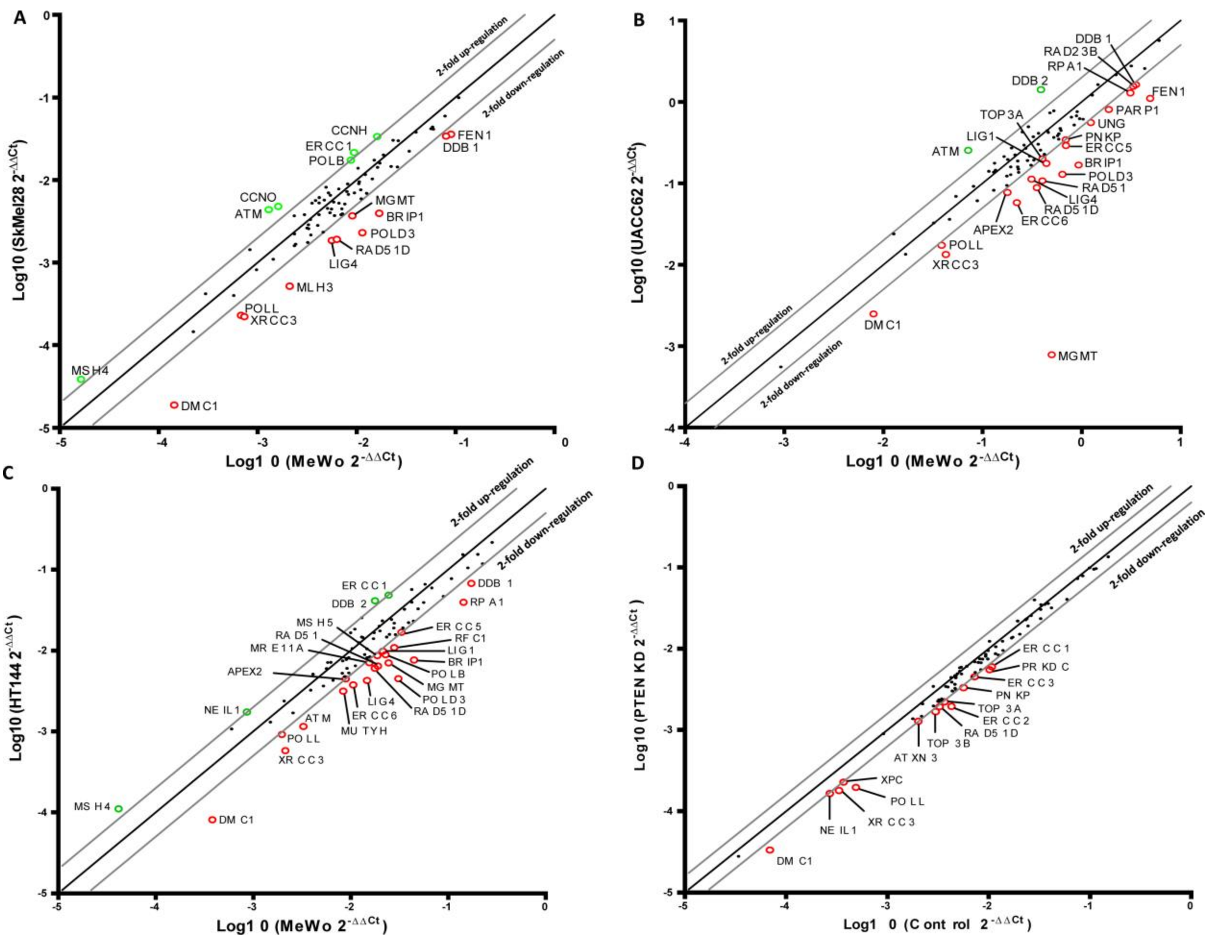

Supplementary Figure S1: Scatter plots indicate up- and down-regulation of DNA repair mRNA expression in PTEN deficient SkMel28 (A), UACC62 (B), HT144 (C) and PTEN knockdown MeWo cells (D) compared to PTEN proficient MeWo cells. Red circle shows genes that are two-fold or more down-regulated and green circles show genes that are two-fold or more up-regulated. See also results section and supplementary table S5.

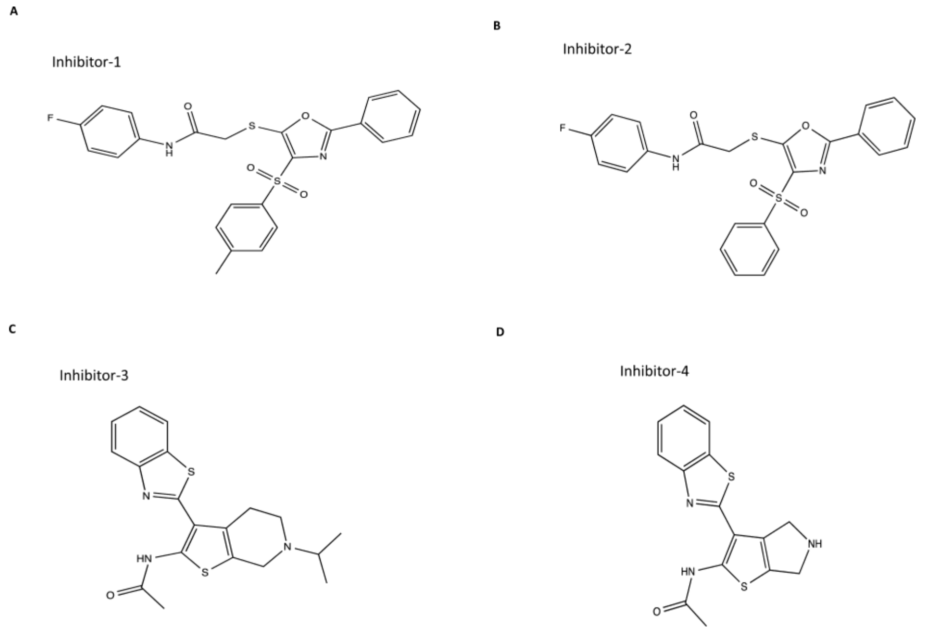

Supplementary Figure S2: Chemical structures of APE1 inhibitors are shown here. APE1 inhibitors investigated here are highly potent and specific for APE1. They do not bind to DNA and have no activity against *E. coli* endonuclease IV (a functional homolog with no sequence or structural homology to APE1). IC_{50} for APE1 endonuclease activity inhibiton in purified protein fluoresence based biochemical assays are as follows; inhibitor 1= 0.2µM, inhibitor 2= 0.1µM , inhibitor 3= 2µM and inhibitor 4=3.3µM . See methods for details.

Supplementary Table S1: Tumour and patient characteristics.

Tumour or patient characteristic	Number or median (% or range)
Sex:<br>M<br>F	 92 (48.2)<br>99 (51.8)
BMI	26.9 (18.1 – 43.2)
Breslow thickness, mm, median (range)	2.8 (0.8 – 14.0)
Mitotic rate, per mm^2, median (range)	5.0 (0.0 – 83)
Ulceration:<br>Yes<br>No<br>Missing	 79 (41.4)<br>85 (44.5)<br>27 (14.1)
Vascular invasion:<br>Yes<br>No<br>Missing	 26 (13.6)<br>151 (79.1)<br>14 (7.3)
Tumour regression<br>Yes<br>No<br>Missing	 22 (11.5)<br>113 (59.2)<br>56 (29.3)
AJCC staging:<br>I<br>II<br>III -IV<br>Missing	 40 (21.0))<br>129 (67.5)<br>22 (11.5)
Relapse status:<br>Yes<br>No	 106 (55.5)<br>85 (44.5)
BRAF – V600E<br>BRAF – V600K<br>NRAS<br>No mutation<br>Missing	84 (0.44)<br>8 (0.04)<br>38 (0.20)<br>52 (0.27)<br>9 (0.05)
Survival status:<br>Died<br>Alive	 101 (52.9)<br>90 (47.1)
Relapse-free survival, years, median (range)	4.4 (0.005 – 11.6)
Overall survival, years, median (range)	6.4 (0.8 – 11.6)
Age at diagnosis, years, median (range)	57.4 (20.0 – 76.8)

Supplementary Table S2: Associations between *PTEN* and *APE1* mRNA expression and tumour characteristics. P-value is presented.

	**PTEN**	**APE1**
Breslow thickness	0.37	0.16
Mitotic rate	0.68	0.04 **
Ulceration	0.83	0.07
Vascular invasion	0.05 *	0.45

Linear regression was used after log transformation for continuous variables (Breslow, mitotic rate) and logistic regression for dichotomous variables (ulceration and vascular invasion).
*: Lower PTEN level associated with vascular invasion
**: Positive correlation between APE1 and mitotic rate

Supplementary Table S3: Effect of PTEN and APE1 on relapse-free and overall survival in Cox proportional hazard model

Survival	Gene	Log2 transformed		Dichotomous (*)	
		**Pvalue**	**HR (95%CI)**	**Pvalue**	**HR (95%CI)**
Relapse-free	*PTEN*	0.01	0.71 (0.54, 0.93)	0.02	0.64 (0.44, 0.95)
	APE1	0.06	1.96 (0.98, 3.90)	0.005	1.94 (1.22, 3.06)
Overall	*PTEN*	0.21	0.84 (0.61, 1.10)	0.02	0.61 (0.41, 0.91)
	APE1	0.03	2.23 (1.09, 4.54)	0.003	2.02 (1.27, 3.20)

With dichotomised variables the baseline is low expression and with continuous log2 transformation the hazard risk (HR) corresponds to increased or decreased hazard by doubling the expression level.

(*) Variable dichotomisation was based on cut-off values calculated using X-tile software .

Supplementary Table S4. Antibodies used in Western blot analysis

Antigen	Antibody	MW	Source	Catalog no.	Dilution
PTEN	Rabbit mAb	54 kDa	Cell Signaling	9188	1:500
APE1	Rabbit pAb	37 kDa	Novus	NB100-101	1:1000
RAD51	Rabbit pAb	37 kDa	Santa Cruz	sc-8349	1:500
BRCA1	Rabbit pAb	220 kDa	Cell Signaling	9010	1:500
BRIP1	Rabbit pAb	145 kDa	Cell Signaling	4578	1:1000
LIG4	Rabbit pAb	96 kDa	Santa Cruz	Sc-28232	1:200
MRE11A	Rabbit pAb	81 kDa	Cell Signaling	4895	1:1000
XRCC2	Rabbit pAb	34 kDa	Novus	NB120-2367	1:100
XRCC3	Rabbit pAb	38 kDa	Novus	NB100-165	1:1500
XRCC4	Rabbit pAb	56 kDa	Novus	NBP1-31339	1:1000
B-actin	Mouse mAb	42 kDa	Sigma	A2228	1:10000

Supplementary Table S5: DNA repair gene expression profiling in SKMel28, UACC62, HT144 and PTEN knockdown MeWo cell compared to MeWo melanoma cell lines.

	**SkMel28**		**UACC62**		**HT144**		**PTEN KD**	
	**Mean fold change**	**p value**	**Mean fold change**	**p value**	**Mean fold change**	**p value**	**Mean fold change**	**p value**
**Down-regulation**								
RAD51	0.489	**0.008**	0.195	**0.002**	0.340	**0.003**	1.024	0.563
RAD51D	0.238	**0.005**	0.177	**0.003**	0.332	0.074	0.414	**0.004**
TOP3A	0.539	**0.029**	0.342	**0.004**	0.872	0.724	0.430	**0.005**
BRIP1	0.225	**0.002**	0.125	**0.001**	0.170	**0.002**	0.661	**0.026**
DMC1	0.113	**0.000**	0.211	**0.001**	0.167	**0.007**	0.408	**0.038**
LIG4	0.384	**0.005**	0.262	**0.003**	0.251	**0.002**	0.684	0.247
DDB1	0.449	**0.000**	0.336	**0.001**	0.389	**0.030**	0.739	0.061
ERCC5	0.459	**0.014**	0.310	**0.005**	0.501	**0.021**	0.883	0.377
ERCC6	0.721	0.119	0.182	**0.005**	0.352	**0.012**	0.424	**0.007**
LIG1	0.770	0.071	0.292	**0.001**	0.459	**0.028**	0.934	0.550
MGMT	0.398	**0.009**	0.001	**0.001**	0.286	**0.036**	0.745	**0.000**
MSH5	0.473	**0.006**	0.486	**0.011**	0.461	**0.046**	0.853	**0.029**
POLD3	0.236	**0.000**	0.137	**0.000**	0.145	**0.004**	0.946	0.091
RPA1	0.688	0.184	0.285	**0.025**	0.266	**0.001**	0.672	**0.011**
**Up-regulation**								
ATM	4.114	**0.001**	2.418	**0.041**	0.077	0.064	1.009	0.963
DDB2	0.630	0.274	2.686	**0.036**	2.343	**0.017**	0.957	0.601

All significant p values are shown in bold.
